# From Inoperable to Remission: Complete Response in Advanced Thymoma B3 With Immunotherapy—A Case Report

**DOI:** 10.1002/rcr2.70433

**Published:** 2025-12-08

**Authors:** Dharmaning Estu Wirastyo, Isnin Anang Marhana, Alfian Nur Rosyid

**Affiliations:** ^1^ Department of Pulmonology and Respiratory Medicine, Faculty of Medicine Universitas Airlangga ‐ Dr. Soetomo General Academic Hospital Surabaya Indonesia

**Keywords:** case report, pembrolizumab, radiotherapy, surgery, thymoma

## Abstract

Thymoma is a rare thymic epithelial tumour, often detected incidentally on imaging. We report a 30‐year‐old female presenting with cough and chest discomfort, whose evaluation revealed a large anterior mediastinal mass compressing the heart and superior vena cava. Histopathology confirmed type B3 thymoma. The patient underwent thymectomy with residual disease, followed by adjuvant radiotherapy and prolonged pembrolizumab immunotherapy. Serial imaging demonstrated marked tumour regression, with residual metabolically inactive tissue on PET scan, consistent with non‐viable remnants. This favourable outcome is notable given the incomplete (R2) resection, where prognosis is typically poor. The case illustrates the effectiveness of a multimodal approach incorporating immune checkpoint blockade in advanced thymoma, an area with limited clinical evidence. Reports on pembrolizumab use after incomplete thymectomy remain limited in the global literature, and this case highlights its potential role in extending disease control and improving outcomes in aggressive thymoma.

## Graphical abstract

This case report describes a 30‐year‐old female with advanced type B3 thymoma managed through a multimodal approach combining surgery, radiotherapy and pembrolizumab immunotherapy. The treatment achieved significant tumour reduction and a complete response on follow‐up imaging, highlighting the potential role of immunotherapy in improving outcomes for aggressive thymoma. Chest imaging. (A) Pre‐treatment chest x‐ray (May 2022) showing mediastinal mass (9.8 × 7.2 × 8.1 cm) compressing the heart and SVC; (F) A Thymoma mass measuring (2.2 × 1.1 × 1.3 cm) post‐completed multimodal treatment chest x‐ray without new mass formation.

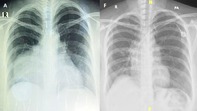

## 1 Introduction

Thymoma, a rare thymic epithelial tumour, is the most common primary tumour in the thymus, with an unknown aetiology and incidence of 0.13 per 100,000. Often asymptomatic, thymomas are usually discovered through chest x‐rays. Thymomas can recur and metastasise. Surgery, often combined with radiation, chemotherapy, or immunotherapy, is the preferred treatment, with ongoing research on immunotherapy's role [1]. There remains a scarcity of thymoma case reports undergoing multimodal therapy, particularly with pembrolizumab immunotherapy alone.

## 2 Case Report

**FIGURE 1:**
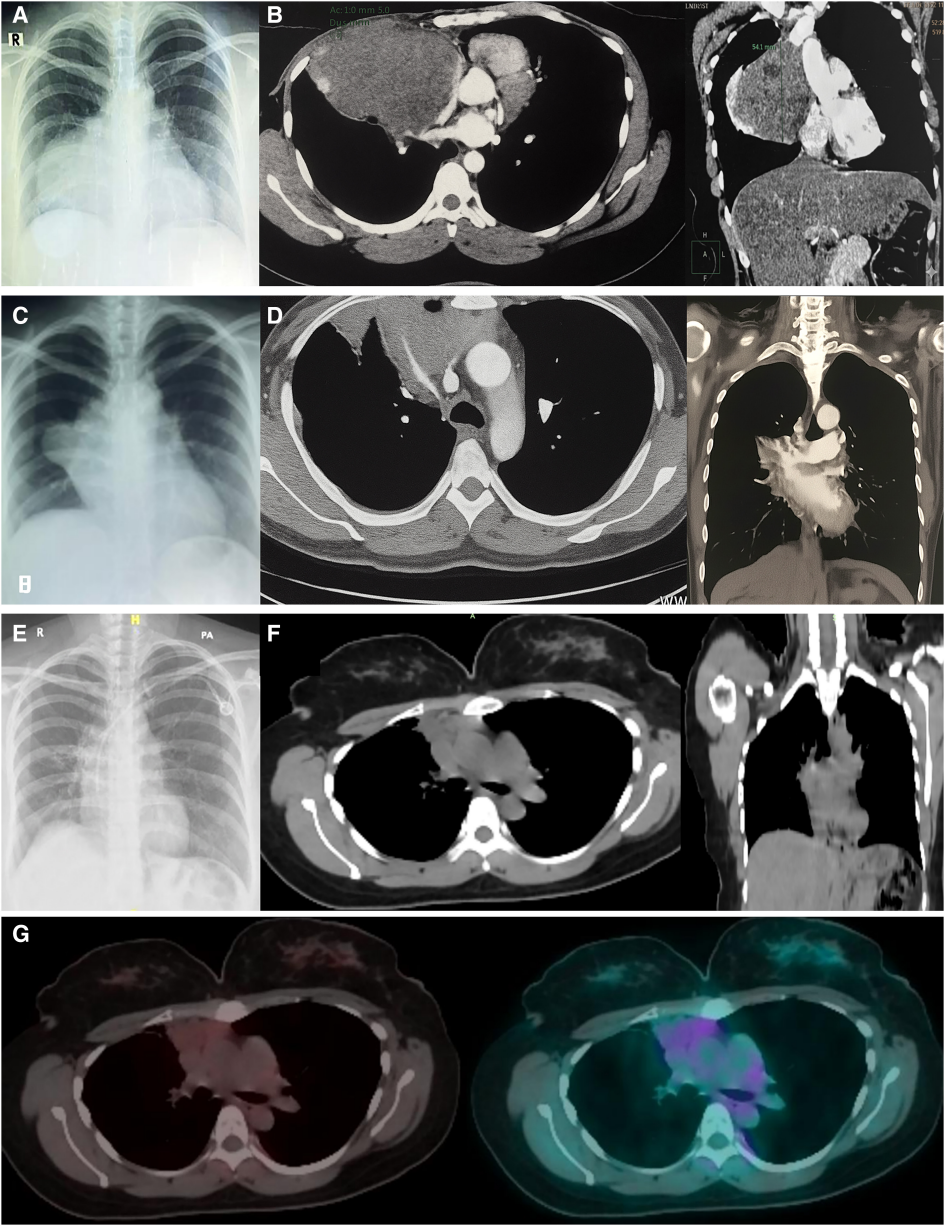
Chest imaging. (A) Pre‐treatment chest x‐ray (May 2022) showing mediastinal mass (9.8 × 7.2 × 8.1 cm); (B) pre‐treatment CT revealing a large anterior mediastinal mass (9.8 × 7.2 × 8.1 cm) compressing the heart and SVC; (C) post‐resection chest x‐ray at one‐month follow‐up showed marked reduction of the mediastinal mass (7.5 × 4.8 × 7.7 cm); (D) post‐resection CT showing residual mass (7.5 × 4.8 × 7.7 cm) with pericardial lesion; (E) chest x‐ray showing a thymoma mass measuring 2.2 × 1.1 × 1.3 cm after three‐month follow‐up completion of multimodal treatment, including surgical resection, radiotherapy, and pembrolizumab; (F) CT scan showing a residual lesion measuring 2.3 × 1.0 × 1.3 cm with no evidence of new mass formation after three‐month follow‐up completion of multimodal treatment; (G) post‐completed immunotherapy PET scan at six‐month follow‐up revealed an irregular‐margined (2.2 × 1.1 × 1.3 cm), likely representing non‐viable tumour residue. No lesions are seen in the lymph nodes or other organs suspicious for additional malignancy.

A 30‐year‐old female presented with cough and chest discomfort for 1 month, with physical examination showing decreased breathing sound at the right hemithorax. The patient's mother was diagnosed with breast cancer in 2021. In the same month, the chest x‐ray demonstrated mediastinal enlargement in the right hemithorax (Figure 1A), and CT imaging confirmed the presence of a large anterior mediastinal mass measuring 9.8 × 7.2 × 8.1 cm with cystic components compressing the heart and superior vena cava (Figure 1B). The patient has been diagnosed with an anterior mediastinal tumour, suspected to be a thymoma.

Following thymectomy, repeat chest x‐ray showed a clear reduction in the mediastinal mass (Figure 1C) at one‐month follow‐up, and postoperative CT demonstrated a smaller mixed lesion in the right pericardium, measuring 7.5 × 4.8 × 7.7 cm (Figure 1D). There was an improvement in the patient's symptoms and complaints after receiving surgical resection, 22 radiotherapy cycles (total dose 55 Gy), and 7 cycles of pembrolizumab were administered.

The multimodal therapeutic approach showed substantial improvement, and a subsequent chest CT revealed further reduction in the mediastinal mass and evidence of pleural thickening. Ongoing immunotherapy is being administered every 3 weeks, with a planned duration of 24 months.

Chest x‐ray performed after 3 months of completion of therapy demonstrated marked improvement compared with the initial findings. A subsequent the chest x‐ray (Figure 1E) and CT scan (Figure 1F), there remains a lesion with irregular margins in the right anterior mediastinum (measuring ~2.3 × 1 × 1.3 cm) which is most consistent with residual non‐viable tumour tissue.

Six months after completing treatment, an evaluation using a PET scan (Figure 1G) revealed an irregular‐margined (2.2 × 1.1 × 1.3 cm), likely representing non‐viable tumour residue. No lesions are seen in the lymph nodes or other organs suspicious for additional malignancy.

## 3 Discussion

Thymomas near thoracic vessels can cause chest symptoms and superior vena cava syndrome. Myasthenia gravis may develop in 30%–40% of cases [2]. The patient had cough, chest discomfort and superior vena cava syndrome grade 0 on initial CT.

Chest x‐rays can detect 45%–80% of thymomas. CT scan (18 HU) were used to differentiate between thymoma and non‐thymoma (90.9% sensitivity and 70% specificity) [3]. The patient's initial CT revealed a solid anterior mediastinal mass with mild post‐contrast enhancement, clear boundaries and multiple cysts, pressing against the superior vena cava. The patient underwent a CT‐guided transthoracic core needle biopsy, yielding a 15 × 10 × 5 mm greyish‐red tissue specimen for histopathological examination, with histopathologic result as thymoma type B3.

Osserman stage IIA‐IV and WHO type B2‐B3 predict the postoperative myasthenic crisis with an incidence of 17.4%, necessitating careful perioperative management [4]. WHO classifies thymic epithelial tumours into types A, AB, B1, B2 and B3. Type B3 thymoma, characterised by lung infiltration, exhibits the lowest 20‐year survival rate at ~40%. Surgery offers a 5‐year survival rate of 61.3% [5]. Complete removal is crucial for prognosis. In this case, an R2 (macroscopic residue) resection addressed a predominantly mass with infiltration into lung, pericardium, superior vena cava, and oesophagus involvement. According to the Masaoka–Koga staging system, these findings are consistent with stage III thymoma. Right thoracotomy allows comprehensive analysis of thymic tumours. Yielding a 195 g yellowish‐brown, nodular tissue for histopathology examines. An incomplete resection (R2 classification) left 10% of the mass, primarily in the right hemithorax, with significant size reduction post‐surgery, continued with radiotherapy and immunotherapy.

Pembrolizumab as immunotherapy has shown promising activity in advanced thymic epithelial tumours, as demonstrated in phase II clinical trials, with response rates around 20%. However, immune‐related adverse events such as myocarditis, myositis and myasthenia gravis occurred more frequently, particularly among thymoma patients, were frequent and sometimes severe, warranting careful patient selection [6, 7]. According to the National Comprehensive Cancer Network (NCCN) Guidelines immune checkpoint inhibitors such as pembrolizumab may be considered for recurrent or refractory thymic carcinoma, although evidence remains limited and clinical discretion is advised. For advanced thymomas, induction chemotherapy before surgical assessment is suggested. Metastasis management includes chemotherapy or immediate surgery, guided by imaging. The NCCN Guidelines favours cisplatin/doxorubicin/cyclophosphamide, with pemetrexed and paclitaxel as second‐line treatments [8]. However, as the patient was a woman of reproductive age who declined chemotherapy due to fertility concerns regarding its potential gonadotoxic effects, immunotherapy provides a potential alternative with a more favourable reproductive safety profile, selected as the preferred treatment approach [9].

Pembrolizumab, targeting PD‐1/PD‐L1, offers promise for thymoma, with some notable severe side effects. High PD‐L1 expression has been reported in up to 71% of thymoma cases, supporting its use [10]. PD‐L1 testing was not conducted in this case due to resource limitations and the need for prompt initiation of treatment. Thymoma patients on pembrolizumab had a 71.4% stable response and a 28.6% partial response [11]. In this case, a 200 mg dose demonstrated good outcomes, including a 20% tumour size reduction after the first cycle.

Postoperative radiotherapy (PORT) is suggested after incomplete thymoma resection starting 3 months post‐surgery. PORT's role post‐complete resection is debated, but it demonstrates a survival benefit by preventing recurrence after incomplete surgeries. In thymoma patients, undergoing radiotherapy offers a 5‐year survival rate of 95% [12]. This case initiated PORT 1‐month post‐R2 resection due to clinical worsening, with a 55 Gy total dose over 22 sessions, achieving improvement post‐five radiotherapy cycles and subsequent immunotherapy.

Thymomas demonstrate a significantly better prognosis, with higher 5‐year survival rates than thymic carcinomas. Treatments include multimodal strategies has contributed to incremental yet clinically relevant improvements in the outcomes of patients with thymic tumours [13].

After undergoing multimodal therapy, the patient was able to regain independence and mass reduction on a 6‐month follow‐up CT. Two years after completing multimodal treatment, the patient demonstrated a complete response.

## Author Contributions

**Dharmaning Estu Wirastyo:** patient evaluation, data collection, manuscript drafting and final approval of the manuscript. **Isnin Anang Marhana:** image selection, writing, review and editing and final approval of the manuscript. **Alfian Nur Rosyid:** references, writing, review and editing and final approval of the manuscript.

## Funding

The authors have nothing to report.

## Consent

The authors declare that written informed consent was obtained for the publication of this manuscript and accompanying images using the consent form provided by the Journal.

## Conflicts of Interest

The authors declare no conflicts of interest.

## Data Availability

The data that support the findings of this study are available from the corresponding author upon reasonable request.
